# An Enhanced Heterologous Virus-Like Particle for Human Papillomavirus Type 16 Tumour Immunotherapy

**DOI:** 10.1371/journal.pone.0066866

**Published:** 2013-06-14

**Authors:** Khairunadwa Jemon, Vivienne Young, Michelle Wilson, Sara McKee, Vernon Ward, Margaret Baird, Sarah Young, Merilyn Hibma

**Affiliations:** 1 Department of Microbiology and Immunology, University of Otago, Dunedin, New Zealand; 2 Department of Pathology, University of Otago, Dunedin, New Zealand; Instituto Butantan, Brazil

## Abstract

Cervical cancer is caused by high-risk, cancer-causing human papillomaviruses (HPV) and is the second highest cause of cancer deaths in women globally. The majority of cervical cancers express well-characterized HPV oncogenes, which are potential targets for immunotherapeutic vaccination. Here we develop a rabbit haemorrhagic disease virus (RHDV) virus-like particle (VLP)-based vaccine designed for immunotherapy against HPV16 positive tumours. An RHDV-VLP, modified to contain the universal helper T cell epitope PADRE and decorated with an MHC I-restricted peptide (aa 48–57) from the HPV16 E6, was tested for its immunotherapeutic efficacy against the TC-1 HPV16 E6 and E7-expressing tumour in mice. The E6-RHDV-VLP-PADRE was administered therapeutically for the treatment of a pre-existing TC-1 tumour and was delivered with antibodies either to deplete regulatory T cells (anti-CD25) or to block T cell suppression mediated through CTLA-4. As a result, the tumour burden was reduced by around 50% and the median survival time of mice to the humane endpoint was almost doubled the compared to controls. The incorporation of PADRE into the RHDV-VLP was necessary for an E6-specific enhancement of the anti-tumour response and the co-administration of the immune modifying antibodies contributed to the overall efficacy of the immunotherapy. The E6-RHDV-VLP-PADRE shows immunotherapeutic efficacy, prolonging survival for HPV tumour-bearing mice. This was enhanced by the systemic administration of immune-modifying antibodies that are commercially available for use in humans. There is potential to further modify these particles for even greater efficacy in the path to development of an immunotherapeutic treatment for HPV precancerous and cancer stages.

## Introduction

Cervical cancer is the second most common cause of cancer in women worldwide [1]. 'High risk', oncogenic human papillomavirus (HPV) types are the primary etiological agents of cervical cancer[2]. The predominant HPV type globally is type 16. DNA from HPV 16 is detectable in more than 50% of all cervical tumours [3]. Cervical cancer has well-defined pre-cancer and cancer stages [4] and is an attractive target for prophylactic and therapeutic vaccination because its etiology is known. Prophylactic virus-like particle (VLPs) vaccines (Cervarix, Gardasil) containing the L1 capsid protein of high risk HPV16 and 18 and adjuvant have been developed. The vaccines generate strong antibody responses with > 98% protection against HPV16 and 18 in naïve individuals [5] [6] [7]. Although HPV VLP vaccines are highly efficacious prophylactically, it is well recognized that they have no therapeutic efficacy [8], [9]. The current vaccines therefore do not address the burden of disease of those with current or previous exposure to HPV16 or 18, nor does vaccination improve health outcomes for women with HPV tumours.

VLPs are engineered by expressing and assembling viral capsid proteins into structures that are immunologically 'comparable' to natural virions, however their immunologic potential as a vaccine carrier is compromised if there is pre-existing immunity to the VLP. Rabbit haemorrhagic disease virus RHDV (*Calciviridae*) is a *Lagovirus* first observed in rabbits 1984 in China [10]. RHDV is a positive-strand RNA virus with a capsid comprised of 180 monomeric units of the 60 kDa capsid protein (VP60), which assemble into 90 dimers to form a 40 nm, *T = 3* icosahedral structure [11], [12]. The RHDV-VLP are replication deficient, but are morphogenically and antigenically identical to the parent virus[13]. In addition to being an effective prophylactic vaccine in rabbits [14], RHDV-VLP can act as vehicles for delivery of heterologous antigens, tolerating both the introduction of foreign antigenic sequences onto the N-terminus of VP60 without compromising particle formation [15] and chemical conjugation of antigenic epitopes to the VLP scaffold [16]. We have previously demonstrated that RHDV-VLP are phagocytosed by both murine and human antigen presenting cells (APC) and that the heterologous VLP-associated antigens can be presented to and recognized by CD8^+^ T cells to generate anti-tumour activity [17]–[19]. As RHDV is an exclusive pathogen of rabbits, anti-RHDV immunity is non-existent within the human population.

Helper T cells, which recognise peptide presented in the context of MHC class II, make a critical contribution to the adaptive immune response both to virus infection and to tumours, producing cytokines and enhancing CTL function both indirectly and directly. Universal helper T cell peptides are capable of binding MHC class II and activating helper T cells with a wide range of specificities and MHC backgrounds. PADRE is a 13-amino acid (aa) peptide that is not naturally occurring (i.e. non-natural) but is designed to have a high affinity for multiple DR alleles in human and mouse [20]. PADRE is potent inducer of human T cell proliferation [21], providing help for CD8^+^ cytotoxic T cells [20]. It can also bind murine I-A^b^ MHC class II molecules, so is applicable to a murine tumour model. The broad activation properties of PADRE make it highly attractive for incorporation into a VLP and its efficacy in enhancing an anti-tumour response will be tested here.

HPV precancerous lesions are characterized by an infiltration of regulatory T cells (Treg) [22]. Tregs, which co-express CD4 and CD25, are immune suppressive, secreting regulatory cytokines such as TGFβ and IL-10 (α chain of the IL-2 receptor) [23]–[25]. The effectiveness of therapeutic vaccines for HPV may be reduced by pre-existing Tregs, which potentially can be overcome by delivering an anti-CD25 antibody to lyse Tregs via the complement pathway.

The cytotoxic T lymphocyte-associated protein 4 (CTLA-4) functions to dampen the T cell response. CTLA-4 is expressed transiently on activated CD4^+^ and CD8^+^ T cells and constitutively on Tregs [26]. Like the co-stimulatory molecule CD28, CTLA-4 binds CD80 and CD86 on antigen presenting cells, but with a higher affinity. On binding, CTLA-4 signaling increases the threshold for T-cell activation, inhibiting T cell responsiveness. Antibody blocking of CTLA-4 inhibits negative regulation of effector T cells.

In this study we test the immunotherapeutic efficacy of an RHDV-VLP surface-decorated with an HPV16 E6 peptide and the potential to enhance its effectiveness by its use in combination with antibody therapy. There was an E6-independent reduction in tumour area in mice vaccinated with the RHDV-VLP in a therapeutic tumour-challenge model, which we attribute to a non-specific immune stimulatory effect of the RHDV-VLP. When a modified E6-RHDV-VLP incorporating PADRE was used, tumour growth was further delayed in an E6 peptide dependent manner. The concomitant administration of PC61 antibody treatment to deplete Tregs cells additionally improved the immunotherapeutic efficacy, as did the use of an anti-CTLA-4 treatment to block the negative regulatory signals through the CTLA-4 pathway. The PADRE-containing heterologous RHDV-VLP carrier is designed to easily translate to other virus or tumour targets, simply by surface-coupling the relevant epitope(s) for the target in question.

## Materials and Methods

### Generation of recombinant RHDV-VLP and RHDV-VLP-PADRE

To insert the PADRE peptide sequence onto the N-terminus of the RHDV VP60 gene, the 87 bp primer PADRE_F (5′-TAGATCTAAAATG **GCCAAGTTCGTGGCTGC** **CTGGACCCTGAAGGCTGCCGCT**GGAGGTTCGGAGGGCAAAGCCCGTGCAGCGCCGCA-3′), was designed (start codon underlined). This primer contains a Bgl II site (AGATCT) for cloning purposes, the PADRE coding sequence (AKFVAAWTLKAAA) (DNA sequence in bold) and a flexible linker (GGS) at the 5 prime end of the VP60 sequence. The VP60 gene was amplified using the PADRE_F primer and an internal VP60 reverse primer (5′-CCAGTCACTACGGCATA-3′) by PCR extension, inserted into pAcUW51 under the control of the AcMNPV p10 promoter and used to generate the recombinant baculovirus by homologous recombination in Sf21 insect cells [27]. The original VP60 [27] and PADRE.VP60 recombinant baculoviruses were plaque-purified twice before expression of the VLP proceeded. RHDV-VLP and RHDV-VLP-PADRE were generated in Sf21 suspension cultures infected with the VP60 or PADRE.VP60 expressing recombinant baculovirus respectively, at a multiplicity of infection of 1.0 and purified by ultracentrifugation on a CsCl gradient, as previously described [19].

The quantity of purified VLPs was calculated following spectrophotometry (extinction at 280 nm) and theoretical molecular weight. Equivalent amounts of protein were run on a 10% SDS-PAGE gel, the protein bands excised and submitted to the Otago Centre for Protein Research for trypsin digestion and mass spectrometry analysis using MALDI-TOF/TOF to confirm the presence of the VP60 and PADRE peptide sequences. Assembly of VLPs was confirmed by electron microscopy at the Otago Centre for Electron Microscopy, University of Otago. Purified VLP was fixed onto carbon-coated grids using 2% phosphotungstic acid negative stain pH 6.8 and viewed under a Philips CM100 Transmission Electron microscope.

### Attachment of the HPV E6 peptide onto the VLP surface

To attach the peptide sequence (EVYDFAFRDL) of the MHC class I restricted E6 epitope (aa 48–57) from HPV type 16 [28] to the surface of the VLP, a cysteine was added to the N-terminus of the peptide to create an available thiol group. A biotin marker added to the C-terminus of the peptide to allow it to be readily detected.

Five milligrams of purified VLP in 0.1 M Na_3_PO_4_ pH 7.2, 0.15 M NaCl was reacted with a 10-fold molar excess of sulfo-SMCC (Thermo Fisher Scientific, Auckland, NZ) for 1h at RT to add an available maleimide group onto the VLP. At the completion of the reaction, the sample was dialysed (10 kDa molecular weight cut off) to remove excess unreacted sulfo-SMCC. Five milligrams of the maleimide-activated VLP was then reacted with a 10-fold molar excess of the E6 peptide (JPT Peptide Technologies, Berlin, Germany) for 1 h at RT to react with the thiol group introduced on the peptide. Unreacted peptide was removed by dialysis.

To confirm the E6 peptide was incorporated onto the VLP, 4 µg of the reaction was run on a 10 % SDS-PAGE gel, transferred onto PVDF membrane, reacted with 0.05 µg/ml streptavidin HRP (Sigma) and detected with SuperSignal West Pico substrate (Thermo Fisher Scientific, Auckland, NZ).

### In vivo tumour treatment experiments

Specific pathogen free female C57BL/6 mice (12 weeks old) were obtained from the Hercus Taieri Resource Unit, University of Otago, New Zealand. All animal experiments were performed according to protocols approved by the Animal Ethics Committee, University of Otago (AEC 41/09). TC-1 tumour cells expressing HPV16 E6 and E7 were a kind gift from Dr. T.C. Wu (John Hopkins University, Baltimore, MD, USA) [29]. Mice (eight per group) were inoculated with 1×10^5^ TC-1 cells subcutaneously (s.c.) on the right flank. Nine days later, mice were vaccinated subcutaneously adjacent to the tumour with 100 µg/mouse of E6-VLP-PADRE, VLP-PADRE, VLP or PBS s.c., followed by a boost with the same dose one week later. Mice were monitored every 2–3 days and tumours were measured using digital calipers until the tumour size was ≥150 mm^2^, at which time the humane endpoint was met and mice were euthanized.

### In vivo antibody treatment

CD4^+^ CD25^+^ regulatory T cells were depleted by intraperitoneal (i.p.) injection of 170 µg/mouse of PC61 [30]. CTLA-4 blockage was performed by i.p. administration of 100 µg/mouse of anti-CTLA-4 antibody (IgG2b; clone 9D9) or an isotype control antibody, IgG2b (clone MPC-11) at day 9, 11 and 13 post tumour inoculation [31]. All antibodies were obtained from BioXCell (West Lebanon, NH, USA).

### Detection of PADRE-specific proliferation and IFNγ secretion in vitro

Mice were immunised with RHDV-VLP or RHDV-VLP-PADRE as described and spleens were harvested 7 days following the second immunisation. Splenocytes were co-cultured with bone marrow derived dendritic cells (DC) that had been cultured for 6 days in DMEM containing 5% fetal calf serum and 20 ng/ml recombinant GM-CSF (Life Technologies, USA) then pulsed for 24 h with PADRE peptide or with unpulsed control DC. Splenocytes and DC were cultured at a ratio of 20∶1 for 72 h, at which time supernatants were harvested and tritiated thymidine added to the cells. Cells were harvested 16 h later and the amount of incorporated thymidine measured using a Wallac Micro-beta counter. Levels of IFNγ were measured by ELISA. Briefly, plates were coated with 50 µl/well of 1 µg/ml anti-mouse IFNγ (BD Biosciences, NJ), washed then incubated with 100 µl samples or IFNγ standards, washed, incubated with 100 µl of 1 µg/ml biotinylated anti-mouse IFNγ (BD Biosciences, NJ), washed and incubated with 100 µl streptavidin-horse radish peroxidase (BD Biosciences, NJ), then incubated with tetramethyl benzidine substrate (Life Technologies, NJ), the reaction stopped with H_2_SO_4_ and the signal read at 450 nm.

### Detection of E6-specific T cells in peripheral blood

Blood was obtained from the tail vein and red cells were lysed by incubating in 144 mM NH_4_Cl in 17 mM Tris-HCl, pH 7.5 for 5 min at 37°C. Cells were resuspended in FACS buffer (PBS, pH 7.2 containing 1% BSA and 0.1% sodium azide) and stained with Alexa 488-conjugated anti-CD8α mAb (BD Pharmingen) and allophycocyanin-conjugated H-2K^b^ E6_48–57_ (EVYDFAFRDL) tetramer [32](NIH Tetramer Facility, Atlanta, GA, USA) for 30 min at 4°C. Cells were washed and resuspended in FACS buffer and samples were acquired using a FACSaria (Becton Dickinson, CA, USA). Data were analysed using Flow-Jo software (Tree Star, Ashland, USA).

### Statistical analysis

Statistical analysis was carried out using the Mann-Whitney U test for comparisons between groups. Comparisons of tumour area were made at day 44 for all groups except the PBS control group, which was the latest time at which no mice had been euthanized. For comparisons with the PBS control group, day 33 was taken, being the latest time at which no mice had been euthanized. Survival curve analysis was performed using the Mantel-Cox Log-rank test. All statistical analysis was carried out with Prism 5.0 (GraphPad Software, CA, USA).

## Results

### Internal incorporation of PADRE into the RHDV-VLP

**Figure 1:**
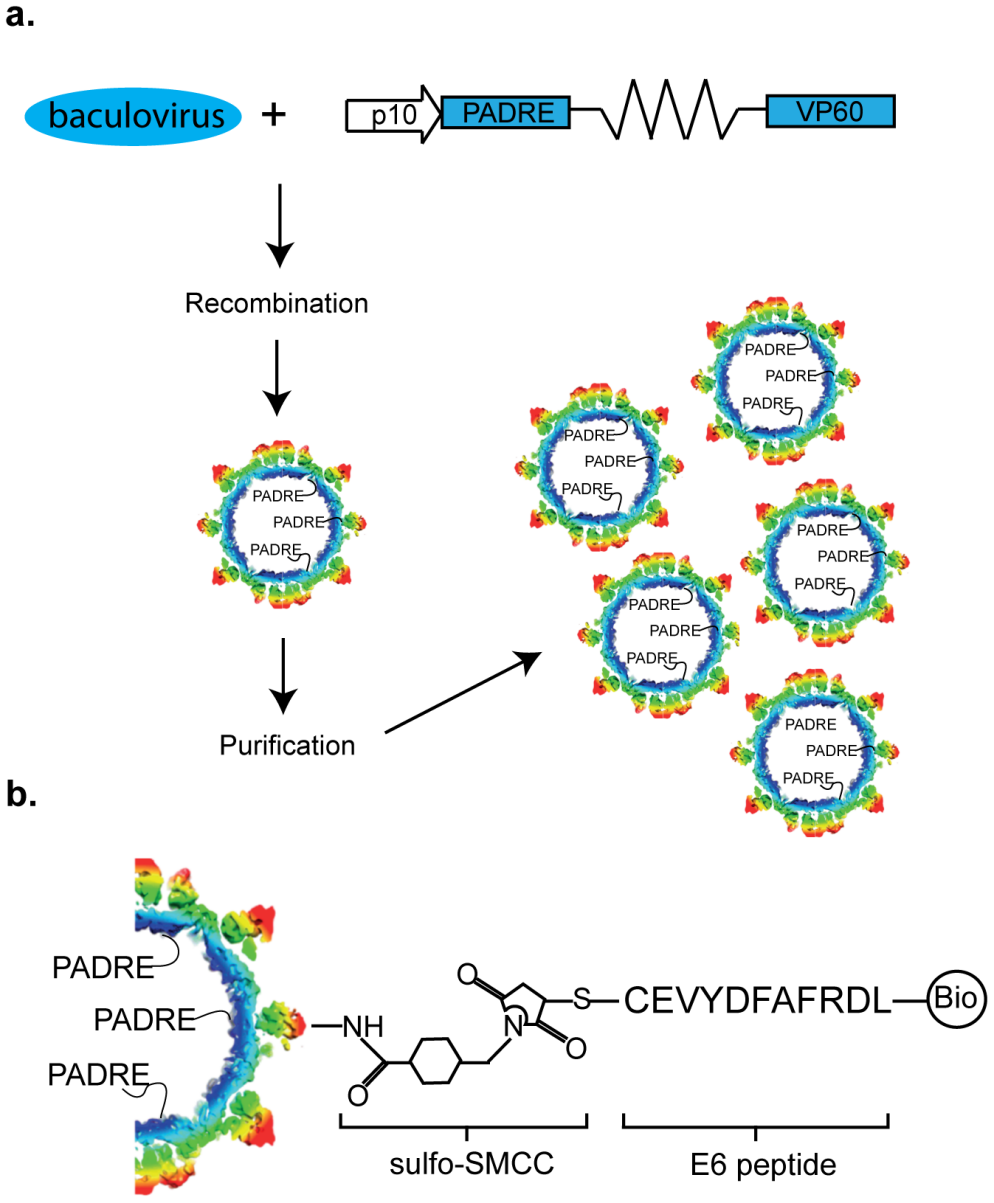
The design and construction of the peptide- and PADRE-modified RHDV-VLP. (a) The VP60 gene modified with a sequence encoding the N-terminal PADRE and flexible linker was recombined into baculovirus in insect cells. Following expression, the RHDV-VLP were purified by CsCl density gradient ultracentrifugation. (b) Diagram of the VLP shell showing the attachment of the PADRE peptide to the internal surface of the VLP by PCR extension and the external attachment of the biotinylated E6 peptide, with the additional N-terminal cysteine, (C-EVYDFAFRDL) by way of a heterobifunctional linker, sulfo-SMCC.

The purpose of this study was to determine the anti-tumour efficacy of HPV16 E6-coupled RHDV-VLP and to establish the effects of the inclusion of PADRE and co-administration of immunotherapeutic antibodies on the anti-tumour response. The RHDV-VLP-PADRE was designed so that the translated PADRE peptide would be on the internal face of the VLP, leaving the surface free for modification with target-specific epitopes. The previously reported 8.1Å cryo-transmission electron microscope structure of the RHDV-VLP shows the N-terminus of VP60 is on its internal face [33]. Based on those data, a plasmid containing the PADRE sequence fused to the N-terminus of the VP60 sequence, via a flexible linker, was constructed (Fig. 1a).

**Figure 2:**
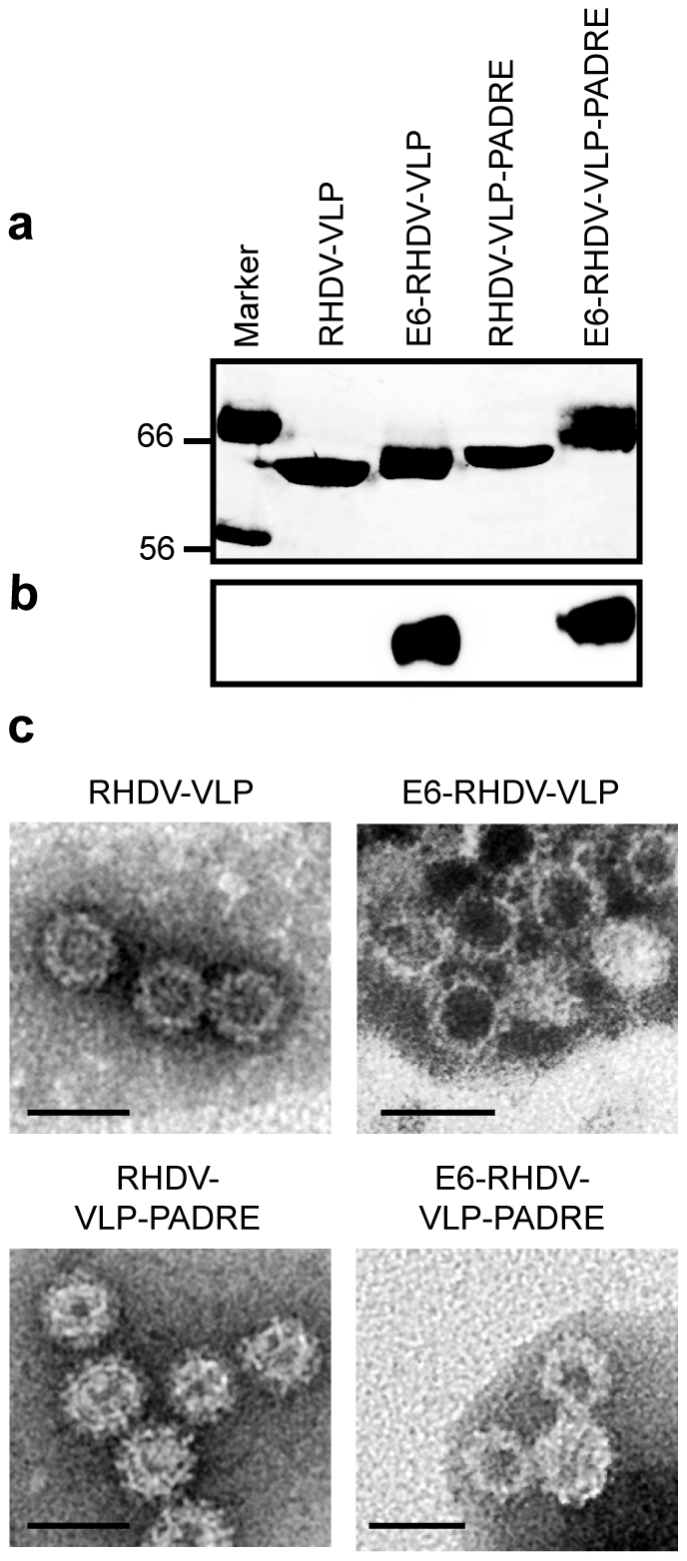
VLP purification and coupling of the E6 peptide. (a) 8% SDS-PAGE and corresponding western blot (b), probed with streptavidin-HRP, of purified RHDV-VLP and RHDV-VLP-PADRE uncoupled or coupled with the biotinylated E6 peptide. Lane 1, Marker (kDa); 2, RHDV-VLP (2 µg); 3, E6-RHDV-VLP (4 µg); 4, RHDV-VLP-PADRE (2 µg); 5, E6-RHDV-VLP-PADRE (4 µg). Electron microscope analysis of purified RHDV-VLP, E6-RHDV-VLP, E6-RHDV-VLP-PADRE and RHDV-VLP-PADRE, confirming assembly (c), clockwise from top left, Bar  = 50 nm.

RHDV-VLP and RHDV-VLP-PADRE were generated by expression of the RHDV VP60 or the PADRE modified VP60 capsid protein in baculovirus. Spontaneously assembled VLPs were purified on a CsCl gradient and analysed by SDS-PAGE to confirm their purity (Fig. 2a). A 60 kDa VP60 band was detected when RHDV-VLP were analysed by SDS-PAGE analysis and a size shift consistent with the addition of the PADRE sequence (1.6 kDa) was detected when RHDV-VLP-PADRE was analysed. The sequence FVAAWTLKAAAGGSEGK (Mascot Database Shapiro-Wilk W test for identity: *P*<0.05), which includes the PADRE sequence, the flexible linker (GGS) and the start of the VP60 sequence (EGK), was identified by mass spectrometry. Electron microscopy was used to show that the modification of the N-terminus of VP60 did not affect the ability of the RHDV-VLP to assemble (Fig. 2c). All RHDV-VLP preparations were visually similar in their appearance and were comparable to previously described RHDV-VLP [27].

### External incorporation of E6 peptide on RHDV-VLPs

Therapeutic strategies for HPV have focused on the major oncogenes, E6 and E7, both of which are expressed by the H-2^b^ restricted TC-1 tumour cell line used in this study. A number of HPV16 E6 and E7 MHC class I restricted epitopes have been described for a range of backgrounds. We chose the E6_48-57_ peptide, one of the more widely used E6 immunodominant MHC I restricted epitopes for this study. The cysteine modified and biotinylated E6 peptide was chemically attached to the external surface of the VLP via the cysteine, using an amine/maleimide linker (Fig. 1b). To confirm attachment of the biotinylated E6 peptide to the external surface of the RHDV-VLP or the RHDV-VLP-PADRE, peptide-coupled reactions were run on an SDS-PAGE gel and a western blot was carried out for the detection of the biotinylated E6. Up to two primary amines are available per VP60 monomer, as determined using NHS coupled with a Dylight 633 fluorescent label (data not shown). A size shift of the VP60 monomer, consistent with the E6-biotin peptide having bound, was identified on the E6 coupled RHDV-VLP and the RHDV-VLP-PADRE following SDS-PAGE (Fig. 2a). The E6-RHDV-VLP and E6-RHDV-VLP-PADRE signals following western blot for detection of biotinylated E6 peptide were comparable (Fig. 2b) and following densitometry analysis had similar intensities (E6-RHDV-VLP: 2.3×10^3^ v. E6-RHDV-VLP-PADRE: 2.4×10^3^ intensity x mm^2^).

### Vaccination with E6-RHDV-VLP delays tumour growth in a therapeutic tumour model

**Figure 3:**
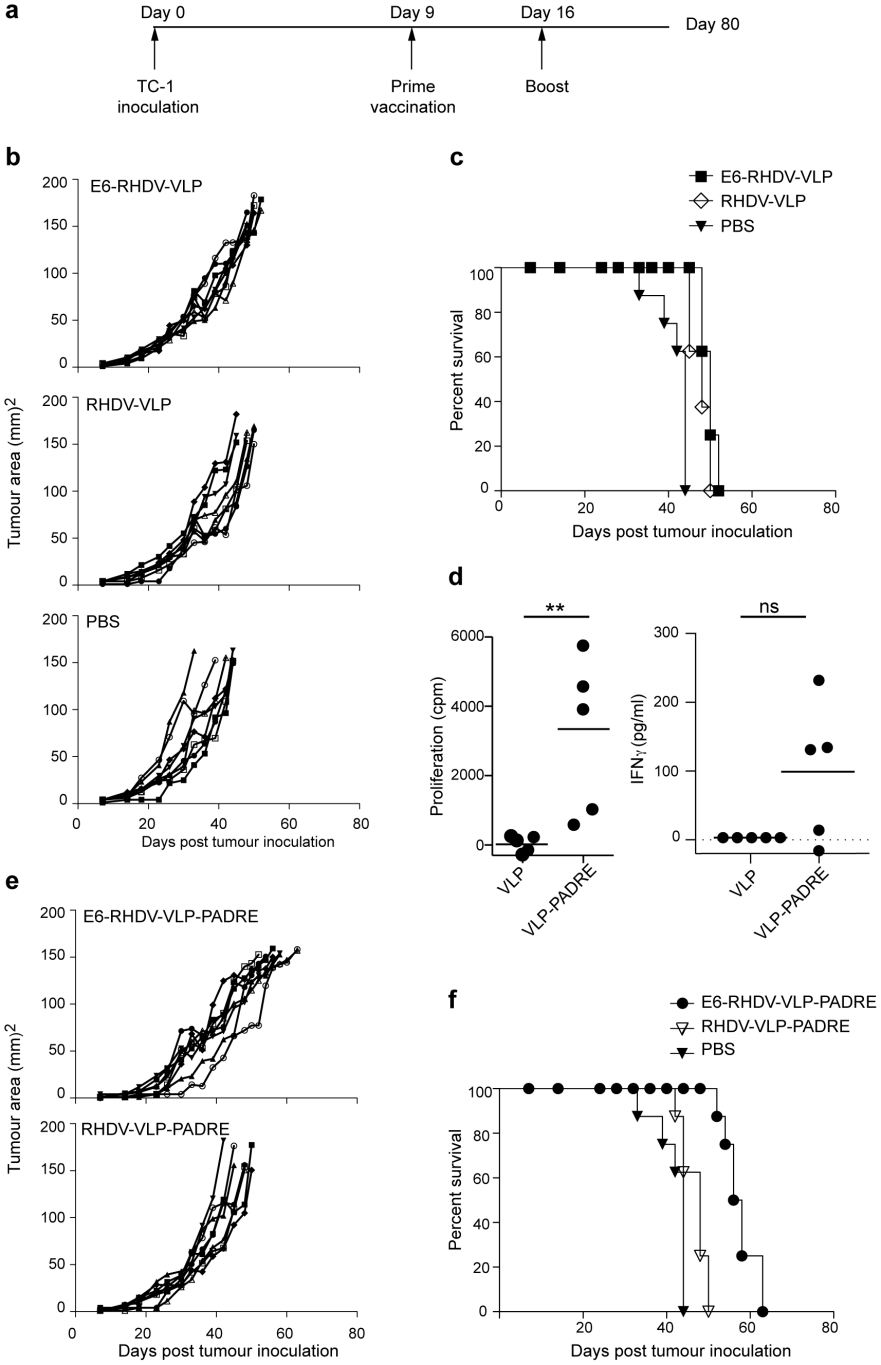
E6-RHDV-VLP-PADRE immunisation reduces tumour burden and increases survival in mice with pre-existing TC-1 tumours. (a) C57BL/6 mice (eight per group) were inoculated with 1×10^5^ TC-1 tumour cells/mouse s.c., vaccinated nine days later followed by a second vaccination after one week. Tumour area (b) and Kaplan-Meier survival curves (c) for mice vaccinated with E6-RHDV-VLP, RHDV-VLP or PBS. T cell proliferation and IFNγ secretion was measured following *in vitro* stimulation of splenocytes with PADRE from mice immunized with RHDV-VLP or RHDV-VLP-PADRE (d). Tumour area (e) and Kaplan-Meier survival curves (f) for mice vaccinated with E6-RHDV-VLP-PADRE, RHDV-VLP-PADRE or PBS immunized controls.

VLP are highly efficacious prophylactically and have potential to be effective carriers therapeutically, both for virus infection and for tumour treatment. The therapeutic efficacy of immunisation with E6-RHDV-VLP against a pre-existing subcutaneous tumour was tested in this study *in vivo* in a mouse model. The model involved injection of C57BL/6 mice with a moderate burden of 1×10^5^ syngeneic E6 and E7 expressing tumour cells (TC-1 cells), which were allowed to establish for nine days prior to vaccination. At that time, mice were vaccinated and boosted s.c. with E6-RHDV-VLP with an interval of one week between doses (Fig. 3a). Control mice were vaccinated similarly, but with RHDV-VLP alone or with PBS. As early as 33 days after TC-1 injection it was necessary to euthanize the first of the PBS control mice, having a tumour with an area ≥150 mm^2^ (Fig. 3b). Although there was no significant difference in tumour size between the RHDV-VLP with or without E6 peptide and the PBS control group at day 33, immunisation with the E6-RHDV-VLP prolonged the median survival by 6 days (Fig. 3c), compared with the PBS control immunized mice (M-C test; *P*<0.0002). Surprisingly, the RHDV-VLP median survival was also increased, extended by 4 days compared with the PBS control group (M-C test; *P*<0.0002) and there was no significant difference in survival between E6-RHDV-VLP and RHDV-VLP immunized mice. It appears that the E6-RHDV-VLP has a modest immunotherapeutic effect that is contributed to by an adjuvanting effect of the RHDV-VLP.

### Incorporation of PADRE increases the efficacy of the E6-RHDV-VLP

We questioned if increased activation of helper T cells, which contribute to the adaptive immune response by secreting cytokines that influence CD8^+^ T cell function [34], [35], would enhance the anti-tumour response. We chose to incorporate a universal helper T cell epitope (PADRE) to activate helper T cells, because of its reported effectiveness and broad applicability. PADRE specific proliferative responses and IFNγ secretion (Fig. 3d) was detected *in vitro* from splenocytes obtained from mice immunized with RHDV-VLP-PADRE and restimulated *in vitro* with PADRE but not from RHDV-VLP immunized and PADRE restimulated splenocytes. The inclusion of E6 peptide on the RHDV-VLP-PADRE led to a reduction in tumour area by around 30% (M-W U test; *P*<0.002) at day 44 (Fig. 3e). The median survival time was increased by 13 days (M-C test; *P*<0.001) in the E6-RHDV-VLP-PADRE immunized mice compared to the PBS control mice (Fig. 3f), whereas the survival of RHDV-VLP-PADRE mice was comparable to the RHDV-VLP mice, with a four-day extension of median survival compared with the PBS control group. The significantly increased median survival in the E6-RHDV-VLP-PADRE immunized mice by 9 days compared to the RHDV-VLP-PADRE mice (M-C test; *P*<0.0001) indicates an E6-specific anti-tumour affect, which contrasts with what we observed in the absence of PADRE.

### The therapeutic efficacy of E6-RHDV-VLP-PADRE is enhanced by PC61 antibody treatment

**Figure 4:**
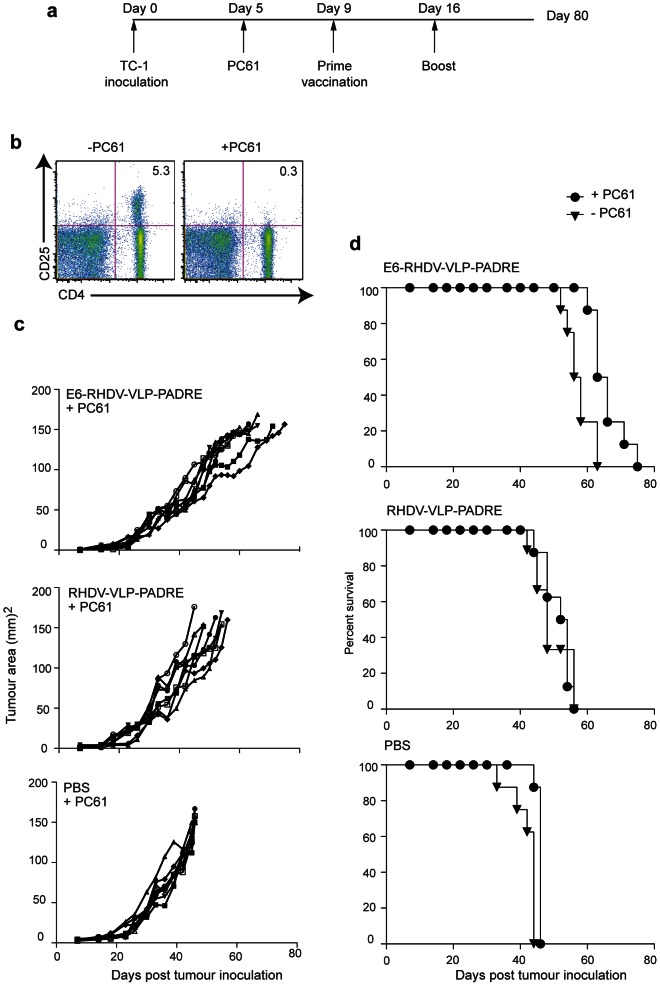
The administration of PC61 further improves the efficacy of E6-RHDV-VLP-PADRE. (a) Schematic diagram depicting the schedule of tumour administration, PC61 injection and the prime and boost vaccination. (b) Flow cytometric analysis for CD4^+^ and CD25^+^ T cells four days following CD25 depletion by injection of 170 µg PC61 ip. Inguinal lymph node cells from an untreated mouse (left panel) and a treated mouse (right panel). (c) Tumour area of mice (eight per group) vaccinated with E6-VLP-PADRE, VLP-PADRE or PBS following administration of PC61. (d) Kaplan-Meier analysis of mice vaccinated with E6-VLP-PADRE, VLP-PADRE or PBS, each with or without PC61 treatment.

There is a significantly increased frequency of FoxP3^+^, CD25^+^ Tregs at the lesion or tumour site in patients with HPV-induced CIN and cervical cancer as well as in other types of cancer including ovarian, lung and breast cancer [36]–[38]. Tregs can be systemically depleted by intravenous delivery of an antibody to CD25 (PC61) in humans and mice. We tested Treg depletion by PC61 administration to determine if it would improve the therapeutic outcome of E6-RHDV-VLP-PADRE immunization in the TC-1 tumour model. Tumour cell administration and vaccination with E6-RHDV-VLP-PADRE, RHDV-VLP-PADRE or PBS was carried out with the additional administration of PC61 four days prior to the primary vaccination (Fig. 4a). Treg depletion in the lymph nodes was validated by flow cytometry and was consistent with previously reported PC61 treatment using this protocol (Fig 4b) [30]. In mice treated with PC61 the mean tumour area following E6-RHDV-VLP-PADRE immunisation was significantly reduced by around 40 % at day 45 (M-W U test; *P*<0.002) compared with PC61-treated mice immunised with RHDV-VLP-PADRE (Fig. 4c) and was around half of that of PC61-treated mice injected with PBS alone (M-W U test; *P*<0.002). PC61 treatment extended median survival to 64.5 days with the E6-RHDV-VLP-PADRE (Fig. 4d), compared with a median of 53 days for the RHDV-VLP-PADRE PC61-treated mice (M-C test; *P*<0.0001) and 46 days with the PC61-treated PBS control mice (M-C test; *P*<0.0001). In comparison to untreated mice, PC61 treatment improved the median survival time of E6-RHDV-VLP-PADRE immunised mice by 7.5 days (M-C test; *P*<0.003). Administration of PC61 also increased the median survival of the RHDV-VLP-PADRE group (five days: M-C test; *ns*) and the PBS control group (two days: M-C test; *P*<0.001), when compared with the respective untreated groups. This was expected, as it has been reported that administration of PC61 alone is sufficient to increase survival in tumour models [39].

### Anti-CTLA-4 treatment reduces the tumour burden and improves survival of mice vaccinated with E6-VLP-PADRE

**Figure 5:**
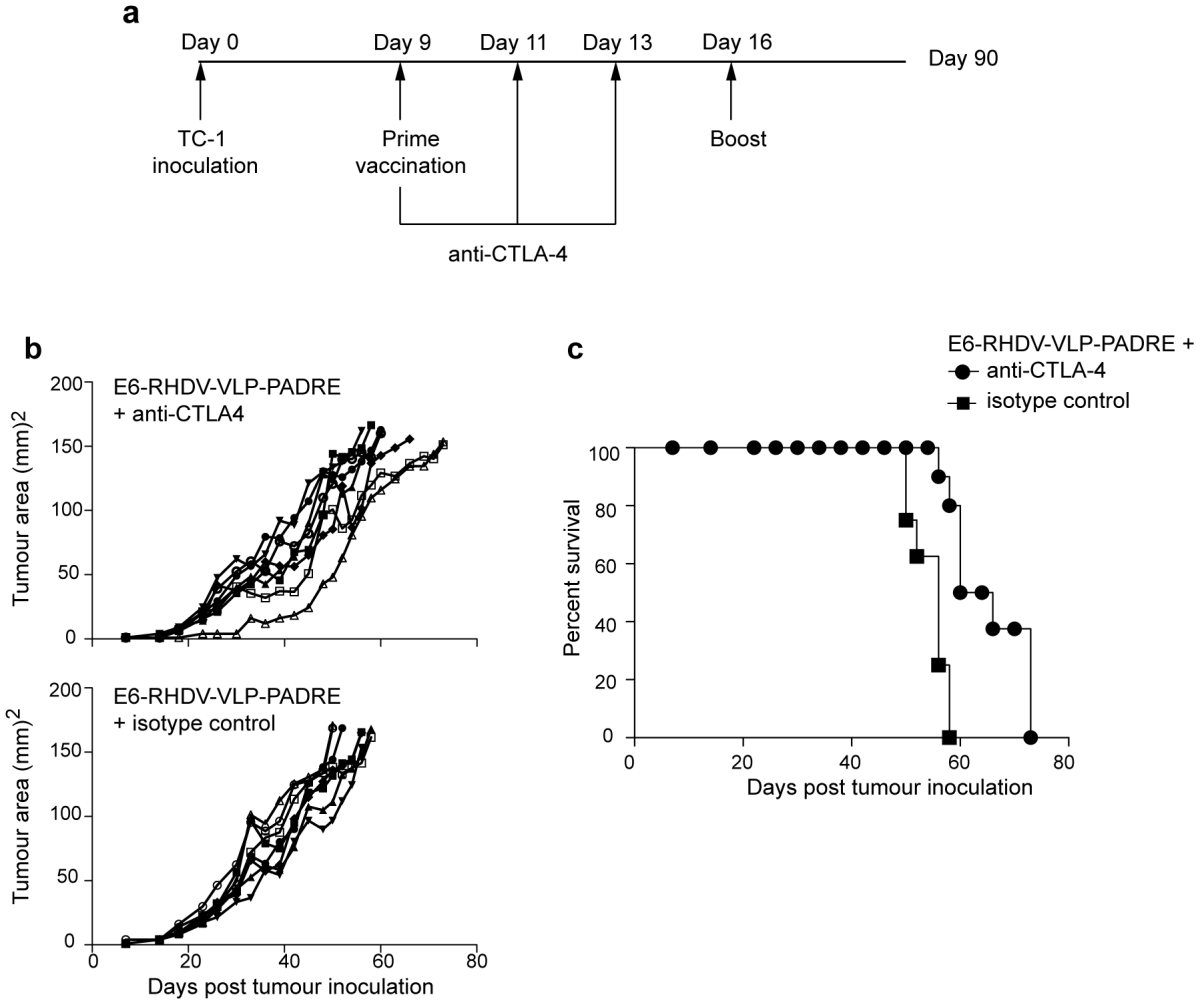
Anti-CTLA-4 blockade enhances the anti-tumour efficacy of E6-RHDV-VLP-PADRE immunisation. (a) The schedule for tumour challenge, anti-CTLA-4 administration, prime and boost vaccination. Tumour area (b) and Kaplan-Meier analysis (c) of the mice (n = 8/group) vaccinated with E6-VLP-PADRE with anti-CTLA-4 treatment or E6-VLP-PADRE with an isotype control antibody administered in place of the anti-CTLA-4 antibody.

Targeting the CTLA-4 signaling pathway has, in other studies, yielded promising results in overcoming suppressive immunological responses against tumours. On that basis we proposed that anti-CTLA-4 antibody therapy in combination with the E6-RHDV-VLP-PADRE vaccination would enhance the anti-tumour response to HPV. To test if anti-CTLA-4 antibody treatment increased the efficacy of the immunisation regimen, anti-CTLA-4 or an isotype control antibodies were administered to mice with pre-existing tumours on the same day as the primary vaccination, then twice more at two day intervals (Fig 5a). We found that the tumour area was reduced by around a third at day 44 in the E6-RHDV-VLP-PADRE vaccinated mice treated with anti-CTLA-4 (Fig. 5b), compared with E6-RHDV-VLP-PADRE vaccinated mice treated with an isotype control antibody (M-W U test; *P*<0.005). The median survival was increased by 7 days (M-C test; *P*<0.0005) by using anti-CTLA-4 treatment in combination with vaccination, compared with treatment with the isotype control (Fig 5c). The systemic administration of anti-CTLA-4 antibody therefore enhanced the therapeutic anti-tumour effect afforded by E6-RHDV-VLP-PADRE. When the immunotherapeutic efficacy of PC61 and anti-CTLA-4 treatment in conjunction with E6-RHDV-VLP-PADRE immunization was compared, both treatments were comparable in their ability to reduce tumour area (M-W U test; *ns*) and enhance survival (M-C test; *ns*).

**Figure 6:**
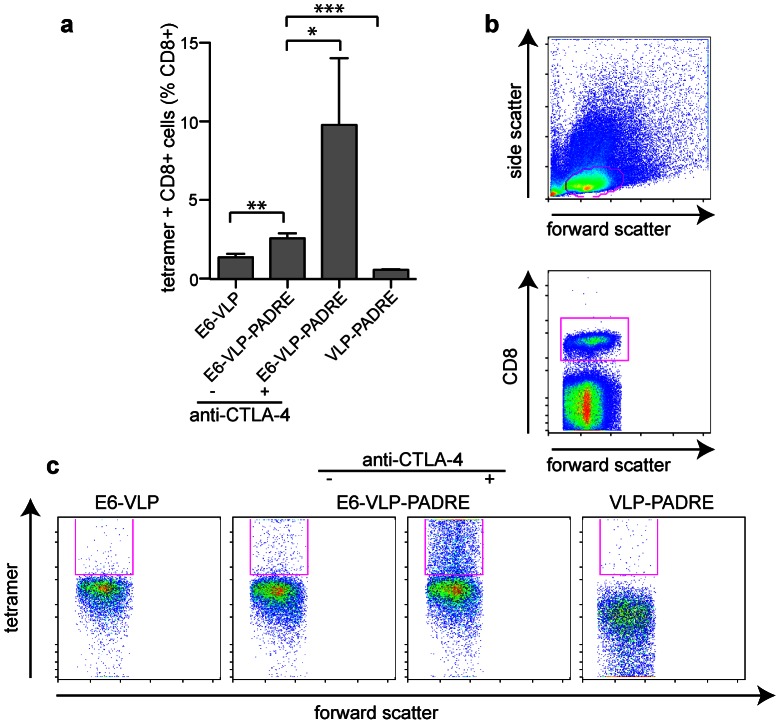
Analysis of the E6-specific CD8^+^ T cell response to immunization with CTLA-4 treatment. (a) Peripheral blood from mice immunized with E6-RHDV-VLP, E6-RHDV-VLP-PADRE, untreated or treated with anti-CTLA-4 antibody, or RHDV-VLP-PADRE (n = 8/group) was analysed for E6-specific CD8^+^ cells. (b) Lymphocytes were gated based on forward and side scatter and of those events, CD8^+^ T cells were analysed. (c) A representative sample of tetramer staining on CD8^+^ lymphocytes is shown for each of the vaccination groups.

CTLA-4 blocking is reported to promote T cell expansion [40]. In order to determine the effect of anti-CTLA-4 on E6-specific CD8^+^ T cell responses, we measured CD8^+^ T cells specific for the E6 epitope in peripheral blood using a tetramer, four days following the boosting immunization. Only around 1% of the CD8^+^ T cells in the blood were specific for the E6 peptide following immunization with E6-RDHV-VLP (Fig. 6). Incorporation of PADRE into the vaccine doubled the percentage of E6-specific CD8^+^ T cells (M-W U test; *P*<0.01). The anti-CTLA-4 treatment had a marked affect on the percentage of E6-specific T cells in the peripheral blood, which was increased almost fourfold compared with vaccinated mice treated with the isotype control (M-W U test; *P*<0.02). From this we conclude that there was a specific increase in expansion of the CD8^+^ T cells generated by the E6-RHDV-VLP-PADRE following anti-CTLA-4 treatment, which was likely due to antibody blocking of the negative regulatory effects of CTLA-4 on T cell proliferation.

## Discussion

There are approximately 529,000 newly diagnosed cases of cervical cancer each year worldwide, with around a 50% mortality rate [41]. Current treatments for high-grade cervical intra-epithelial neoplasia (CIN) include excisional techniques such as radical hysterectomy, hysterectomy, and knife-cone biopsy, or ablative therapies such ascryotherapy, laser ablation and cold coagulation. Both excisional and ablative approaches are associated with a high probability that disease will reoccur [42], [43]. Thus, there is a continuing need to develop better therapeutic treatments for HPV to effectively eliminate HPV precancerous lesions, without disease recurrence.

In this study we test RHDV-VLP as a platform for therapeutic vaccination against HPV precancerous lesions. We found the RHDV-VLP modified by the addition of the PADRE sequence was stable and structurally similar to RHDV-VLP. Therapeutic administration of RHDV-VLP-PADRE surface-labeled with HPV16 E6 peptide suppressed growth of the TC-1 HPV16 E6 and E7 expressing tumour. Tumour regression typically correlates with Th1 responses and CD8^+^ CTLs [44]. We detected PADRE specific T cell proliferation and IFNγ secreting cells following immunization with the PADRE containing VLP, consistent with this vaccine generating a Th1 response. Tumour survival was also significantly improved by the inclusion of PADRE into the E6-RHDV-VLP. Consistent with this report, immunization with a DNA vaccine containing the sequence encoding PADRE also generated PADRE-specific helper T cells and enhanced the anti-tumour response in the TC-1 tumour model [45].

Immunization with E6-RHDV-VLP-PADRE expanded E6-specific CD8^+^ T cells at a significantly higher frequency than the E6-RHDV-VLP vaccine, supporting a functional role for the PADRE stimulated helper T cells in the expansion of E6 specific CD8^+^ T cells. The detection of increased numbers of E6-specific CD8^+^ CTLs in the periphery after E6-RHDV-VLP-PADRE vaccination correlated with reduced tumour size and lengthened survival time indicating a likely infiltration of E6 specific CD8^+^ T cells into the tumour, as has been reported by others to be necessary for tumour regression [46]. We do recognize that variations in the amount of E6 peptide bound to the VLPs may contribute to the differences in the percentages of E6-specific T cells in the blood when comparing the E6-RHDV-VLP with the E6-RHDV-VLP-PADRE as we were able only to analyse the amounts bound semi-quantitatively by western blot. Irrespective of this proviso, these data clearly show that the E6-RHDV-VLP without PADRE did not have an E6-specific anti-tumour effect. Furthermore, we have previously shown that an RHDV-VLP decorated only with an ovalbumin (OVA) class I restricted epitope not have any efficacy against an OVA expressing B16 tumour and that addition of a class II restricted epitope with the class I epitope was required for anti-tumour response [17]. These data and reports by others [47] show that helper T cells are necessary for an effective anti-tumour response.

Delivery of the RHDV-VLP or the RHDV-VLP-PADRE in the proximity of the tumour modestly but significantly extended the median survival time of mice, suggesting a non-specific anti-tumour effect even without E6 peptide. Other studies support non-specific anti-tumour effects of RHDV-VLP [48] and chimeric HPV VLP [49] when co-delivered with CpG. In contrast, ovalbumin (OVA) RHDV-VLP and CpG vaccinations in an aggressive, fast-growing B16.OVA melanoma model failed to show any activity attributable to the RHDV-VLP [50]. In addition to their ability to self-adjuvant, VLPs have immune stimulatory functions [51], such as induction of dendritic cell (DC) maturation and rapid cytokine secretion on VLP binding [52]. The non-specific anti-tumour activity of the RHDV-VLP that is clearly shown in this study may be attributed to the immune stimulating effects of VLPs and the variability of this observation between studies may be contributed to by factors such as the time of delivery of the VLP relative to tumour induction, the stage and aggressiveness of the tumour, and the proximity of the site of VLP delivery relative to the tumour.

Vaccines that induce Th1 cells promote CTL responses and are reported to reduce the accumulation of Tregs in tumour-draining lymph nodes [53]. However Welters *et al*., (2008) showed that E6/E7 synthetic long peptide vaccination in humans expanded antigen-specific Tregs [54]. TC-1 tumours contain infiltrates of immune suppressive Tregs [30], recruited following secretion of the CCL22 chemokine by tumour cells [36]. Inclusion of PC61 treatment to deplete Tregs in conjunction with E6-RHDV-VLP-PADRE immunization extended survival times by around 50% in the TC-1 tumour model compared to PBS immunized mice, providing evidence of the efficacy of the Treg depletion. This is likely to result from the depletion of suppressive tumour-infiltrating Tregs in the tumour, which is reported to contribute to the ability of the TC-1 tumour to grow in immune competent mice [38] [36]. The depletion of Tregs would also prevent vaccine-induced expansion of this population, should that occur following administration of E6-RHDV-VLP-PADRE. Whether the Tregs are expanded as a result of this vaccine is an interesting question that is yet to be tested.

Administration of anti-CTLA-4 blocks negative regulation of T cell expansion. Consistent with this, we found a greater than threefold increased frequency of E6-specific CD8^+^ T cells following inclusion of anti-CTLA-4 immunotherapy to the E6-RHDV-VLP-PADRE vaccination regimen. We also found the median tumour survival was increased by around 50 % with the inclusion of CTLA-4 treatment. Tuve, *et al*., (2007) found that repeated systemic administration of anti-CTLA-4 had no anti-tumour effect in the TC-1 model and indeed induced autoimmunity [55]. In other models, anti-CTLA4 antibody treatment has been reported to enhance the T-cell mediated tumour rejection of colon carcinoma [56], melanoma [57], prostate cancer [58] in mouse models as well as in human cancer patients [59], although treatment induced autoimmunity can occur [55]. Several studies have focused on tumour-localized rather than systemic administration of anti-CTLA-4 to specifically expand lymphocytes at the tumour site, with some success [55], [60] and this strategy could be applied to the vaccine regimen reported here to further enhance efficacy.

Anti-human CD25 and CTLA-4 antibodies are commercially available and are already in use clinically. In patient trials, effective CTL responses to a tumour antigen were generated following vaccination and anti-CD25 Treg depletion in patients with metastatic cancers [61] [62] and improved survival resulted from inclusion of anti-CTLA-4 treatment with immunization for metastatic melanoma [63]. The commercial availability of these antibodies provides ready availability for their application for the combination treatment using the RHDV-VLPs in humans and advances in tumour-targeted delivery of immunotherapy also have applicability in a refined E6-RHDV-VLP-PADRE vaccine for use against HPV precancerous lesions.

Therapeutic use of the H2-Kb immunodominant E6 epitope tested here has not been widely reported. Huang, *et al*. (2011) used a DNA vaccine where the E6 epitope, β2 macroglobulin and MHC class I heavy chain were linked and showed tumour nodules at reduced numbers in the lung 30 days after i.v. challenge with 1×10^4^ tumour cells three days prior to vaccination [64]. In another study, Hung *et al*., (2007) reported that 40 % of E6-PADRE vaccinated mice had tumours at day 14, the time when 100% of the control group had tumours after subcutaneous challenge with 1×10^4^ TC-1 cells three days prior to a two dose DNA immunization regimen [45]. Compared to those studies, we used tenfold more cells and allowed cells to expand for nine days rather than three and showed similar effectiveness to Hung *et al*., albeit over a longer timeframe. Comparisons of efficacy between studies are difficult due a range of variables between studies, however studies that have reported high therapeutic efficacy in the TC-1 tumour model use full-length E6 [65] or E7 [66] or E6 and E7 epitopes or proteins in combination [67], frequently with adjuvants. We predict that the inclusion of multiple epitopes from both E6 and E7 into the RHDV-VLP-PADRE will improve the anti-tumour efficacy of this vaccine. The RHDV-VLP has the capacity for protein to be coupled to the surface therefore it is feasible that E6, E7 or multiple epitopes from HPV proteins in long peptides could be attached [16]. In addition, there is scope to further modify the E6-RHDV-VLP-PADRE vaccine regimen to improve the anti-tumour efficacy. For example, improved effectiveness of a VLP vaccine in an anti-viral response to vaccinia virus has been reported by inclusion of a TLR9 agonist, CpG, into the vaccine and inclusion into this vaccine may similarly have therapeutic benefits [68]. We have recently reported modification of the RHDV-VLP with alpha-galactosylceramide results in activation of iNKT cells with immune-enhancing effects for the antigen-specific immune response [19]. This potentially could also be applied to the E6-RDHV-VLP-PADRE. Additionally, we found that administering anti-CTLA-4 or anti-CD25 treatment with the E6-RHDV-VLP-PADRE vaccination increased survival. Combinatorial treatment with anti-CD25 and anti-CTLA-4 could be applied to the E6-RHDV-VLP-PADRE vaccination regimen, particularly as a good therapeutic outcome with the antibodies used in combination has been shown in models of mouse melanoma [69], colon carcinoma [70] and previously in the TC-1 HPV model [55].

In this study we show that vaccination with E6-RHDV-VLP-PADRE prolongs survival for a pre-existing HPV16 E6 and E7 expressing tumour. The therapeutic effect was further enhanced by systemic administration of antibodies specific for CD25 or CTLA-4. The benefits of the regimen tested here include the potential ease with which it could be translated for use in humans - the PADRE is functional in humans and antibody therapies for CD25 and CTLA-4 are currently commercially available; the modified RHDV-VLP-PADRE carrier having 'in built' helper T cell activating ability by means of PADRE and that the external decoration of the VLP can be readily modified to accommodate other similar or larger peptides. The application of this technology to HPV immunotherapy would be highly beneficial for the large global burden of disease caused by this virus, however it is anticipated that this system could be easily modified for application to a wide range of other virus infections and tumour types.
